# Origin and pathogenicity variation of *Plasmopara viticola* in China

**DOI:** 10.3389/fmicb.2024.1433024

**Published:** 2025-01-15

**Authors:** Wei Wu, Yuchen Chen, Huimin Huang, Rongfang Li, Bohan Yang, Junli Lv, Ling Yin, Junjie Qu, Shiren Song, Yachun Peng, Peining Fu, Jiang Lu

**Affiliations:** ^1^Center for Viticulture and Enology, School of Agriculture and Biology, Shanghai Jiao Tong University, Shanghai, China; ^2^Chongqing Research Institute, Shanghai Jiao Tong University, Chongqing, China; ^3^Guangxi Crop Genetic Improvement and Biotechnology Key Lab, Guangxi Academy of Agricultural Sciences, Nanning, China

**Keywords:** downy mildew, pathogenicity test, plant-pathogen interaction, hypersensitive response, *Vitis* species

## Abstract

Grapevine downy mildew caused by *Plasmopara viticola* (Pv) is one of the most devastating diseases of grapevine in China. To understand the origin and pathogenicity of Chinese Pv, a total of 193 single-sporangiophore isolates were obtained from 14 Chinese major viticulture areas. Phylogenetic analyses suggest that Chinese Pv isolates originate from North America and belong to the *P. viticola* clade *aestivalis*. Host range experiments reveal that Chinese Pv are able to infect a wide range of *Vitis* species from different geographic origins, including Eurasian species *Vitis vinifera*, North American species *V. aestivalis*, *V. riparia*, and *V. rupestris*, and East Asian *Vitis* species *V. davidii*, *V. amurensis*, and *V. hancockii.* Analyses of the interactions between Pv isolates and grapevines reveal that the virulence of Pv isolates is correlated with the occurrence time and magnitude of hypersensitive response-mediating leaf necrosis in grape leaves caused by Pv. These understandings of genetic diversity and pathogenicity of Chinese Pv isolate would be useful to develop strategies for controlling grapevine downy mildew spread.

## Introduction

*Plasmopara viticola* [(Berk. and Curt.) Berl. and de Toni] (Pv), an obligate biotrophic oomycete causing grapevine downy mildew, belongs to the family *Peronosporaceae*. Downy mildew is one of the most important grapevine disease threatening berry yield and quality (Gessler et al., 2011; Koledenkova et al., 2022). Pv infects all green organs of grapevines, including inflorescences, fruits, buds, and new tips, however, it primarily damages the leaves. Pv sporangia spread to wet grape leaves by wind and subsequently release zoospores (Kiefer et al., 2004; Gessler et al., 2011). In free moisture on the leaf, the zoospores swim to stomata and germinate hyphae through stomata into the leaf. The infection is established when haustoria are formed to extract nutrients from the host cell. New sporangia generate in infected areas when the temperature is higher than 10°C (Gessler et al., 2011). When sporulation is impeded or leaves are senescent, oospores are produced through sexual reproduction in a heterothallic manner (Gessler et al., 2011; Dussert et al., 2020). After winter, these oospores mature, and then they germinate and form sporangia when exposed to water in spring or summer (Gessler et al., 2011).

Pv originates from North America. It was introduced into Europe and then spread worldwide because of grape material exchange (Fontaine et al., 2021). Recent phylogenetic studies revealed that North American Pv isolates can be categorized into five cryptic species, i.e., *P. viticola* clade *vinifera*, *P. viticola* clade *aestivalis*, *P. viticola* clade *riparia*, *P. viticola* clade *quinquefolia,* and *P. viticola* clade *vulpine* (Rouxel et al., 2013; Rouxel et al., 2014). Each Pv cryptic species has a different host range. For example, *P. viticola* clade *aestivalis* can infect *V. vinifera*, *V. labrusca*, and *V. aestivalis* cultivars, while *P. viticola* clade *vulpine* infects only *V. vulpine* cultivars (Rouxel et al., 2014).

Pv was originally endemic to North America, where it infected wild *Vitis* species. Then it was probably introduced into Europe in the 1870s, along with American grape rootstock, which was resistant to phylloxera, one of the most destructive insect pests of cultivated grapes (Lafon and Bulit, 1981). In Europe, Pv was first observed in France in 1878 and spread rapidly throughout Europe (Millardet, 1881). Grapevine downy mildew was subsequently found in Italy in 1878, in Germany in 1879, in Greece and Turkey in 1881, in South Africa in 1907, and in Australia in 1917 (Gessler et al., 2011). The first record of grape downy mildew in China was in Xinjiang Province in 1899 (Lei, 2004). Today, downy mildew is observed in major grape-growing areas in China, especially in the eastern and southern regions of the country (Yin et al., 2014).

Grapevines belong to the genus *Vitis* in the family *Vitaceae* and are among the main global horticultural crops. There are approximately 60 inter-fertile wild species in the genus *Vitis* (This et al., 2006). According to their geographical distribution and ecological characteristics, grapevines can be divided into three populations, i.e., the North American, the Eurasian, and the East Asian populations. There are 28 species in the North American populations, including *V. riparia*, *V. labrusca*, *V. aestivalis,* and *V. rupestris*. The East Asian population comprises of more than 30 species, including *V. amurensis*, *V. davidii*, *V. hancockii*, and *V. yeshanensis.* Only one species (*V. vinifera*) represents the Eurasian population, and it is the most widely cultivated grapevine in the world; however, it is highly susceptible to Pv (Gessler et al., 2011).

China is the origin of most East Asian grape populations, and has a large amount of wild grape resources. Many Chinese wild grape varieties show high resistance to Pv, including *V. pseudoreticulata* and some varieties of *V. amurensis* (Wan et al., 2007; Yu et al., 2012; Fu et al., 2020). China is the largest producer and consumer of grapes worldwide, with diverse climatic conditions and abundant cultivated varieties (Kaya et al., 2023; Li et al., 2016). These characteristics have likely shaped Pv populations and genetic diversity in China. Yin et al. (2014) studied the high genetic diversity of Chinese Pv populations by simple sequence repeat (SSR) makers. Li et al. (2016) observed high levels of gene flow among populations and long-distance dispersal of Pv in China. However, few studies have been conducted to learn about the virulence diversity across Chinese Pv populations (Li et al., 2016; Yu et al., 2012).

There has been a common interest to understand the origin of the Chinese Pv pathogen. Zhang et al. (2017) found that the current Chinese Pv population is a mixture of endemic and introduced variants. However, Fontaine et al. (2021) reported that the *P. viticola* clade *aestivalis* native to North America is the origin of all invasive grape Pv populations worldwide, including those in China. The studies of Zhang et al. (2017) included evident confusion in the phylogenetic analysis, whereas that of Fontaine et al. (2021) had only 50 samples of Pv from China. Due to such limited research output, the origin of Chinese Pv populations remains controversial. In addition, highly Pv-resistant grapevines are found among some Chinese *Vitis* species, which raises the question whether there is a native Pv population in China. The isolation and identification of Pv isolates in major grape growing areas will help to reveal the origin of Pv in China. The objectives of this study are to investigate the origin and virulence of Chinese Pv populations using phylogenetic analysis and virulence analysis.

## Materials and methods

### Collection and purification of Pv isolates

**Figure 1:**
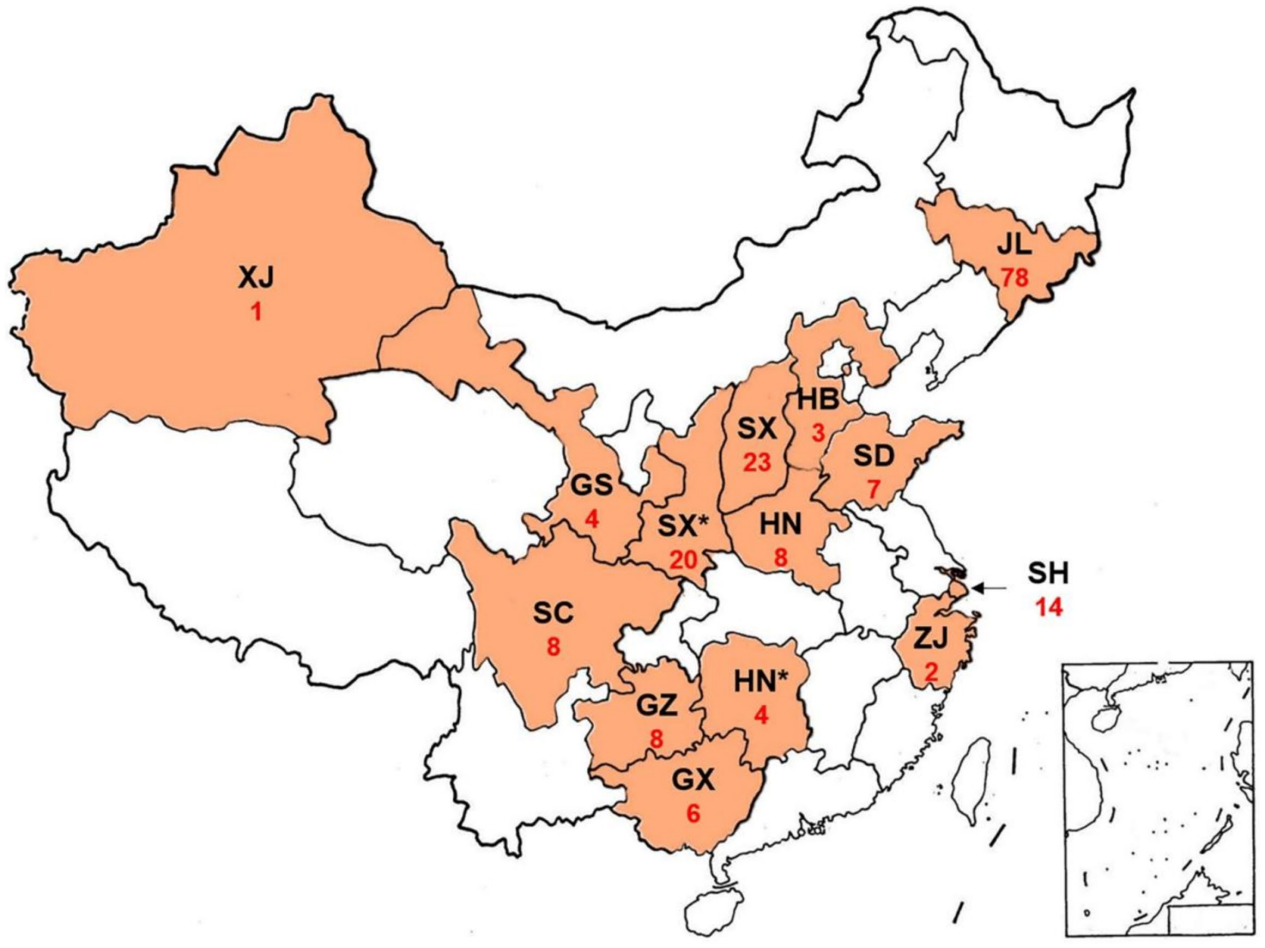
Sampling of *Plasmopara viticola* on wild and cultivated species of the family *Vitaceae* in China. GS, Gansu Province; GX, Guangxi Province; GZ, Guizhou Province; HB, Hebei Province; HN, Henan Province; HN*, Hunan Province. JL, Jilin Province; SD, Shandong Province; SC, Sichuan Province; SH, Shanghai Province; SX, Shaanxi Province; SX*, Shaanxi Province; XJ, Xinjiang Province; ZJ, Zhejiang Province; Red numbers, number of obtained isolates.

Pv isolates were collected from naturally infected cultivateds and wild grapevines in the main grape-growing regions of China (Figure 1; Supplementary Table S1.1). In vineyard, at least three infected grape leaves were collected from each grapevine plant, and at least three grapevine plants were sampled for each variety. In the wild, at least two infected grape leaves were collected from each plant.

*Vitis amurensis* and *V. pseudoreticulata* are two wild Chinese species with high resistance to Pv (Wan et al., 2007; Yu et al., 2012; Fu et al., 2020), however, the habitat of *V. pseudoreticulata* is in the densely populated eastern China. Therefore, the habitat of *Vitis amurensis* located in Changbai Mountain was selected for sampling. The Changbai Mountain is a nature reserve with more than 50 kilometers in diameter. The collection route and location of the Changbai Mountain were uploaded to the database of foooooot.com.^1^

Fresh leaves colonized by sporulating Pv and collected from a single grapevine were immediately placed in a sealed plastic bag and then were placed in a cooler for shipment to Shanghai Jiao Tong University. The leaves were washed with sterile water and placed on a wet filter paper in a round Petri dish with a lid (90 mm in diameter). The production of fresh sporangia were induced by incubation for 3 days in a climate chamber maintained at ~60% RH and 25°C (Gessler et al., 2011), under 12 h light (400 μmol s^−1^) /12 h dark cycles (Ningbo Jiangnan Instrument Factory, RXZ-1000A).

To obtain pure isolates, Pv was inoculated onto leaves of greenhouse-grown *V. vinifera* ‘Thompson Seedless’, which is a highly susceptible *V. vinifera* variety (Gray et al., 2005; Xu et al., 2021). Leaves were immersed in 1% sodium hypochlorite for 10 min for surface disinfection and then washed with sterile water three times. A hole puncher (1 cm in diameter) was used to make leaf disks, which were placed on wet filter paper in a Petri dish with a lid. Each Petri dish contained six leaf disks. A single sporangiophore with sporangia was picked up with a pair of sharp forceps under a stereomicroscope(Tuming Optical Instrument Co., ZOOM-650C). A drop of sterile water (about 50 μL) was placed on each leaf disk, and the sporangiophore with sporangia was placed into each drop. Plates with leaf disks were lidded and incubated for 1 day in the same conditions as the field samples. The leaf disks were kept moist by adding water. Excess water was removed by blotting with sterile filter paper. Sparse sporangia began to appear on the leaf disks 3 days post-inoculation (dpi). There were three to four leaf disks that could be successfully infected by Pv, and three of these were selected for isolation. The single-sporangiaphore isolation was repeated two additional times, after that the sample was considered as a purified isolate. Three single-sporangiaphore Pv isolates were obtained from each grape variety from each collection site. Collection sites and host varieties of Pv isolates are listed in Supplementary Table S1.1.

Sporangia of each isolate were collected on a nylon membrane using a vacuum pump as previously reported (Thines et al., 2010) and were stored at-80°C. When the preserved isolates were required for analysis, the sporangia on the nylon membrane were eluted using sterile water and were inoculated onto leaf disks of the greenhouse*-*grown ‘Thompson Seedless’ plants. Leaf disks were incubated as described above for collection and purificationof Pv isolates. At 6 dpi, dense sporangia were eluted using sterile water and were used for further inoculation.

### Identification of Pv isolates

Genomic DNA of Pv-infected leaves was extracted using modified CTAB method (Yin et al., 2010). Pv-infected leaves (about 0.1 g) were ground into powder in liquid nitrogen and transferred to a 1.5 mL tube. Immediately added 600 μL CTAB solution (2% CTAB; 1.4 M NaCl; 0.1 M Tris–HCl, pH 8.0; 0.025 M EDTA.Na_2_, pH 8.0; 2% PVP40; 2% 2-mercaptoethanol) and incubated for 30 min at 65°C. Then, 600 μL of chloroform was added, and the mixture was fully vortexed and centrifuged at 12,000 g for 10 min. Upper clarified liquid was transferred to a new 1.5 mL tube, then equal volume of isopropanol pre-cooled at-20°C was added. The sample was mixed by vortexing and centrifuged at 12,000 g for 10 min. The supernatant was discarded, 1 mL of 70% ethanol was added, and the mixture was centrifuged at 12,000 g for 10 min after storing at room temperature for 30 min. The supernatant was discarded, and 40 μL of sterile water was added after the precipitate was dried.

SSR genotyping was performed to remove putative clones from the set of isolates to be used in further analyses. Sixteen SSR loci selected from previous studies (Gobbin et al., 2003; Rouxel et al., 2012) were used for amplification. The loci with low SSR size polymorphism were discarded, while new loci were added to ensure that at least five loci were genotyped in strains isolated from the same region. Fragment analysis of amplicons was performed using an automated capillary electrophoresis system ABI 3730XL at the Shanghai Map Biotechnology Company. All isolates were genotyped by at least three of the selected loci, and isolates collected from the same collection site were genotyped by at least five loci (Supplementary Table S1.2). The Pv isolates were regarded as the same clone if they were collected from the same sampling site and contained the same SSR allele sizes. One clone was stored for each genotype for further research. Genomic DNA of water-treated ‘Thompson Seedless’ leaf was extracted and used as a negative control. The SSR primers used in SSR genotyping were listed in Supplementary Table S1.9.

### DNA polymorphisms and phylogenetic analysis

Four loci were amplified and sequenced for phylogenetic analysis, i.e., the internal transcribed spacer region 1 (*ITS*), a fragment of the *β*-tubulin gene (*Tub*), a fragment of the actin gene (*Act*), and a fragment of the cytochrome b gene (*Cytb*). These loci were previously used to characterize cryptic Pv species (Rouxel et al., 2014; Rouxel et al., 2013; Fontaine et al., 2021; Zhang et al., 2017). Specific primers were used for DNA amplification as described in a previous study (Rouxel et al., 2014; Rouxel et al., 2013). The volume of the PCR reaction was 40 μL, comprising of 20 μL fast-pfu PCR superMix (Catalog # SB-221, Share-bio, Inc., Shanghai, China), 1.6 μM primer pairs, and 1 μL (about 200 ng) of genomic DNA. Thermocycling was performed under the following conditions: 95°C for 3 min, 35 cycles of 98°C for 10 s, 56°C for 10 s, 72°C for 30 s, followed by 72°C for 5 min. All amplifications were performed using a T100 Thermal Cycler (Bio-Rad Laboratories, Inc.). PCR products were purified using a column DNA gel extraction kit (Catalog # B518131, Sangon Biotech, Shanghai, China). Sequencing of PCR products was conducted at the Beijing Genomics Institute. The PCR products with unspecific sequcing results were constructed into the cloning vector pLB (Catalog # VT206-01, Tiangen, Beijing, China), and then five positive clones were randomly selected for sequencing. The sequence classification of each isolate is listed in Supplementary Tables S1.3, S1.4. The genomic DNA extracted from water-treated ‘Thompson Seedless’ leaf served as a negative control.

Data from each locus was analyzed separately using DNAMAN version 7.0 (Lynnon Biosoft Company), and all sequences were aligned using ClustalX software (Thompson et al., 1997). Aligned sequences of each gene region and of the concatenated dataset were analyzed using DnaSP v. 5.10.01 (Librado and Rozas, 2009) to identify DNA polymorphisms.

All sequences were collapsed into haplotypes for phylogenetic analysis, and heterozygous were separated into two haplotypes in phylogeny (Supplementary Tables S1.3, S1.4). Phylogenies were inferred using both maximum likelihood (ML) methods and maximum parsimony (MP) methods implemented in Mega X (Kumar et al., 2018). The MP tree was produced using the Tree-Bisection-Regrafting algorithm (Nei and Kumar, 2000) with search level 1 in which the initial trees were produced by the random addition of sequences (1,000 replicates). The corresponding sequences of *P. halstedii* were used as the outgroups. Sequences of North American Pv isolates were downloaded from GenBank and used as references (Supplementary Table S1.4).

### Host range and virulence test

All grapevine leaves used for virulence tests were obtained from the Grape Germplasm and Breeding Research Vineyard of Shanghai Jiao Tong University, 800 Dongchuan Road, Minhang District, Shanghai, China (121° 250′ 34.13′′ E; 31° 010′ 11.15′′ N). A total of 15 different grape varieties or accessions, including two *Muscadinia rotundifolia* varieties (‘Carlos’ and ‘Noble’), two *V. vinifera* varieties (‘Thompson Seedless’ and ‘Cabernet Sauvignon’), three North American *Vitis* varieties (*V. aestivalis* ‘SJTU118’, *V. riparia* ‘SJTU127’, and *V. rupestris* ‘SJTU123’), six East Asian *Vitis* varieties (*V. davidii* ‘Huiliang’, *V. davidii* ‘Ziqiu’, *V. piasezkii* ‘SJTU001’ (Fu et al., 2019a), *V. hancockii* ‘SJTU002’ (Fu et al., 2019b), *V. amurensis* ‘Shuanghong’, and *V. yeshanensis* ‘SJTU004’), and two *Vitis* interspecific hybrids (‘Kyoho’ and ‘Zuohong 1’) were used in the host range test. The information on these grape varieties was listed in Supplementary Table S1.5. *Muscadinia rotundifolia* was used as a control since it belongs to the *Vitaceae* family and is a non-host of Pv. ‘Thompson seedless’ and ‘Cabernet Sauvignon’ are common Eurasian *Vitis* varieties for raisin-making and wine-making, respectively. They were highly susceptible to Pv and used as positive controls. Twenty-eight Pv isolates from major or special grape varieties in various regions were selected to represent Chinese major vine-growing areas in the virulence assay (Table 1; Supplementary Table S1.8). For example, two Pv isolates isolated from *V. amurensis* cultivars and four from wild *V. amurensis* grapevines were used for virulence assays as *V. amurensis* is the main species in Jilin and Changbai Mountains in Jilin, which is the main habitat of wild *V. amurensis* grapevines.

**Table 1:** The number of isolates carrying a given haplotype in different host types.

Loci	Haplotype	GenBank ID	Host types						
			Vv	Va	Vq	*Vs*	Vp	Vd	Hyb
*Act*	*A1*	MW682154		1		1		1	2
	*A2*	MW682155	41	59	3	1	1	4	47
	*A3*	MW682156		1					1
	*A4*	MW682157	9	8				4	2
	*A5*	MW682158	5	7				2	1
	*A6*	MW682159	5	2					
	*A7*	MW682160		1					
*Tub*	*T1*	MW682161	7	1				1	10
	*T2*	MW682162		53	3	1	1	3	61
	*T3*	MW682163	21	4				4	16
	*T4*	MW682164		2					
	*T5*	MW682165		3					
	*T6*	MW682166		4		1		2	
	*T7*	MW682167		15					
	*T8*	MW682168		8					
*ITS*	*I1*	MW652679	50	77	3	1	1	7	52
	*I2*	MW652681	1						
*CytB*	*C1*	MW682169	25	2				4	25
	*C2*	MW682170	10	66	3				15
	*C3*	MW682171		5		1		1	1
	*C4*	MW682172	8						3
	*C5*	MW682173							1
	*C6*	MW682174		1					
	*C7*	MW682175							1

Vv, Vitis vinifera; Va, *V. amurensis*; Vq, *V. quinquangularis*; Vs, *V. sylvestris*; Vp, *V. piasezkii*; Vd, *V. davidii*; Hyb, *Vitis* interspecific hybrids.

The fifth or sixth leaves of grapevines were collected and were disinfected using 1% sodium hypochlorite for 10 min. After disinfection, the leaves were washed thrice using sterile water. A hole puncher (1 cm in diameter) was used to produce leaf disks, which were placed in a Petri dish with a lid (90 mm in diameter) covered and wet sterile filter paper. Each Petri dish contained six leaf disks. The sporangia of Pv isolates to be tested were collected in sterile water and were counted manually using a hemocytometer. The sporangia concentration was adjusted to 100,000 sporangia/mL using sterile water. A 20 μL drop of the sporangia suspension was placed onto the leaf disc, then the leaf disk was incubated at 25°C with a 16 h photoperiod in a climate chamber. Excess water was removed with sterile filter paper at 1 dpi. Photographs of the leaf disks were taken at 6 dpi. The experiment was repeated twice, and at least six leaf disks (one Petri dish) were used per treatment. ‘Thompson Seedless’ leaves grown in the greenhouse were inoculated with the Pv isolates as a positive control, and those inoculated with water were used as a negative control.

According to the *P. viticola* resistance descriptor 452–1 of the International Organization of Vine and Wine^2^ and previous study (Fu et al., 2020), virulence was assessed by sporulation density in the host range test, and mycelial growth in the histological staining. Sporulation density and mycelial growth were visually assessed using the following qualitative ordinal rating scale: not virulent = numerous necrotic spots without sporulation; weakly virulent = sparse necrosis with scattered sporulation; moderately virulent = moderate sporulation with little necrosis; highly virulent = dense sporulation without necrosis. The virulence level of each Pv isolates was assessed based on the results of two independent experiments.

### Histological staining

The infected leaves used in host range analysis were collected for histological staining at 0, 12, 24, 48, and 96 hpi. LJ-Pv18001 had the same virulence as most of the tested isolates, therefore it was selected for histochemical staining. LJ-Pv19056 was selected since it was compatible with *V. amurensis* varieties.

Aniline blue staining was performed according to a previously described method with modifications (Díez-Navajas et al., 2007). The plant material was decolorized at 65°C in decolorizing solution (ethanol and lactic acid in a volume ratio of 1:1) for at least 1 day. The decolorized leaves were washed three times using sterile water. Samples were soaked in staining solution (0.05% aniline blue in 0.067 M K_2_HPO_4_, pH 9.0–9.5) for at least 2 days. The samples were examined and photographed using a fluorescence microscope (Leica DFC450C).

Trypan blue staining was performed as previously described (Tian et al., 2019). The plant material was boiled in 0.25% trypan blue in a dye solution (ethanol, glycerin, phenol and water at a volume ratio of 1:1:1:1) for 5 min. Samples were washed thrice using clean water. The plant material was decolorized in decolorizing solution (saturated chloral hydrate) until the leaves were transparent. The samples were then examined and photographed using a dissecting microscope (Leica M205C).

## Results

### DNA polymorphism of Chinese Pv population

A total of 193 Pv isolates were obtained from different vineyards in 14 provinces of China (Figure 1). The result of DNA polymorphism showed that there were seven *Act* haplotypes (termed *A1* to *A7*) from 189 Pv isolates, eight *Tub* haplotypes (termed *T1* to *T8*) from 191 isolates, two *ITS* haplotypes (termed *I1* to *I2*) from 192 isolates, and seven *Cytb* haplotypes (termed *C1* to *C7*) from 171 isolates (Supplementary Table S1.3). Heterozygosity was found at the *Act* and *Tub* loci of some isolates (Supplementary Table S1.3). Haplotypes of Pv isolates in different regions were also listed in Supplementary Table S1.7. The sequences of the haplotypes obtained were deposited in GenBank: *Act* sequences *A1* to *A7* (MW682154 to MW682160), *Tub* sequences *T1* to *T8* (MW682161 to MW682168), *ITS* sequences *I1* (MW652679) and *I2* (MW652681), and *Cytb* sequences *C1* to *C7* (MW682169 to MW682175).

DNA polymorphisms and diversity of each locus are presented in Table 2. Compared with the other three loci, the *Tub* locus produced the most haplotypes and parsimony informative sites. It also had the highest haploid diversity. However, there was only one non-synonymous mutation in the six variable sites of the *Tub* locus. Seven haplotypes and four parsimony informative sites occurred at the *Act* locus, of which four variable sites were synonymous substitutions. The *Cytb* locus showed the highest nucleotide polymorphism, with seven haplotypes and four parsimony informative sites, and four non-synonymous mutations at seven variable sites. The *ITS* locus had the lowest nucleotide diversity, and only one isolate (LJ-Pv19170) showed a difference at a single site.

**Table 2:** Global polymorphism of the nucleotide alignments of *P. viticola* sequences for the four genomic regions analyzed.

Locus	n^a^	bp^b^	S^c^	nA^d^	PiA^e^	hd^f^	Pi^g^	D^h^
*Act*	189	435	4	7	4	0.4300	0.00164	0.05372
*Tub*	191	531	6	8	6	0.6270	0.00197	0.00092
ITS	192	234	1	2	0	0.0110	0.00005	−0.96038
*Cytb*	171	644	8	7	4	0.5910	0.00304	0.8944

^a^Sample size (n). ^b^Total number of sites (bp). ^c^Number of segregating sites (S). ^d^Number of alleles (nA). ^e^Number of parsimony informative site between alleles (PiA). ^f^Haplotypic (allelic) diversity (hd). ^g^Nucleotide diversity (Pi). ^h^Tajima’s (D).

Among seven *Cytb* haplotypes identified from 171 Pv isolates (Supplementary Table S1.3), the haplotypes C1, C3, and C7 contained mutation G143A that led to the resistance of Pv to the quinone outside inhibiting (QoI) fungicides. Most G143A mutations were found in the isolates from commercial vineyards (60 out of 123), whereas only 4 out of 48 Pv isolates collected from wild species contained this mutation site (Supplementary Table S1.6).

### Phylogenetic analysis of Chinese Pv population

**Figure 2:**
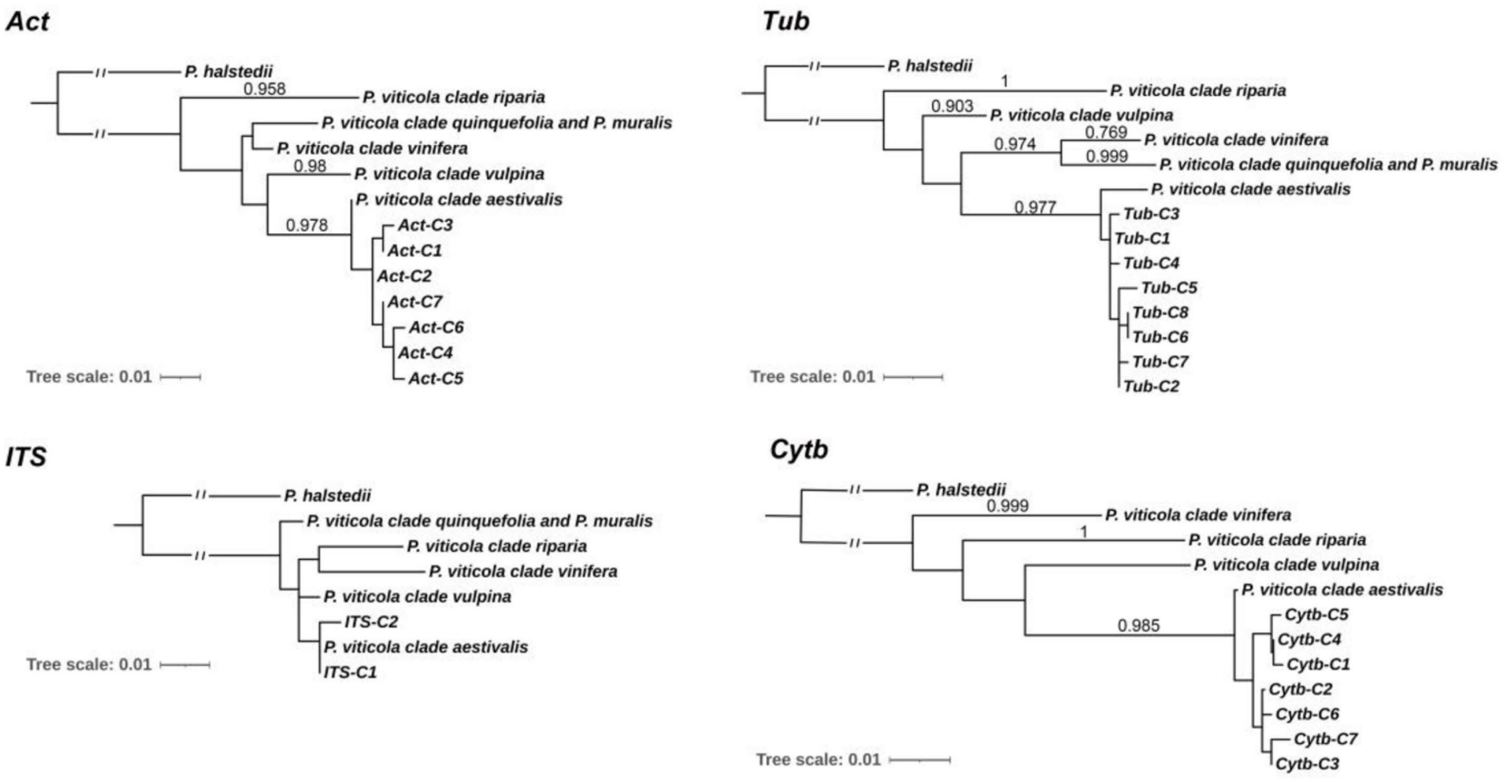
Molecular Phylogenetic analysis of Chinese and North American isolates by Maximum Likelihood method. The percentage of replicate trees in which the associated taxa clustered together in the bootstrap test (1,000 replicates) are shown next to the branches. Bootstrap values are given when superior to 0.700 for both the maximum likelihood and parsimony analysis. Act-C1 to C7, Tub-C1 to C7, ITS-C1 to C2, and Cytb-C1 to C7 are the haplotypes of the Chinese Pv isolates.

To understand the phylogenetic relationships of Pv populations between Chinese and North American, MP phylogenetic trees were constructed based on each of the four independent loci (Figure 2), and the corresponding sequences of *Plasmopara halstedii* were used as outgroups. Consistent with previous reports (Rouxel et al., 2014), all Pv isolates were classified into five lineages, i.e., five cryptic species. The phylogenetic results showed that the Chinese isolates collected from different regions were all assigned to the *P. viticola* clade *aestivalis* (Figure 2). A similar result was also obtained using ML analysis (Supplementary Figure S1). The number of isolates carrying a given haplotype in different host specis and regions were listed in Table 1 and Supplementary Table S1.7.

### Host range and virulence levels of Chinese Pv population

The results of the host range test (Table 3) showed that the Chinese isolates, despite some individual variance, were typically able to infect most *Vitis* species (virulence levels from weakly virulent to highly virulent), including *V. vinifera*, *V. aestivalis*, *V. riparia*, *V. rupestris*, *V. davidii*, *V. piasezkii*, *V. hancockii*, *V. amurensis*, *V. yeshanensis*, and interspecific *Vitis* hybrids. It indicates that the Chinese isolates had a wide host range.

**Table 3:** Results of the virulence assay of 28 *P. viticola* isolates on the grapevines.

Isolates	Host	Location			Grape varieties/accessions												
			Muscadine		Eurasian		North American			East Asian						Hybrid	
			Mr^a^	Mr^b^	Vv^c^	Vv^d^	Vae^e^	Vri^f^	Vru^g^	Vd^h^	Vd^i^	Vp^j^	Vh^k^	Vam^l^	Vy^m^	Hyb^n^	Hyb^o^
LJ-Pv18001	Hyb	Shanghai	N	N	H	H	H	H	L	H	H	H	L	N	H	H	H
LJ-Pv19003	Hyb	Shanghai	N	N	H	H	H	H	M	H	H	H	N	N	H	H	H
LJ-Pv19012	Hyb	Shanghai	N	N	H	H	M	H	M	H	H	H	N	M	H	H	H
LJ-Pv18002	Vq	Guangxi	N	N	H	H	M	H	L	H	H	H	H	M	M	H	M
LJ-Pv18010	Vq	Guangxi	N	N	H	H	H	H	L	H	H	H	H	M	H	H	M
LJ-Pv19017	Va	Jilin	N	N	H	H	H	M	L	H	H	H	H	H	H	M	H
LJ-Pv19029	Va	Jilin	N	N	H	H	H	M	L	H	H	H	H	H	H	H	H
LJ-Pv19035	Va	Jilin*	N	N	H	H	H	M	M	H	M	H	M	H	H	M	H
LJ-Pv19043	Va	Jilin*	N	N	H	H	M	H	M	H	H	H	H	H	H	M	H
LJ-Pv19056	Va	Jilin*	N	N	H	H	H	M	L	H	H	M	H	H	H	H	H
LJ-Pv19068	Va	Jilin*	N	N	H	H	H	H	L	H	H	H	H	H	H	H	H
LJ-Pv19095	Hyb	Shaanxi	N	N	H	H	H	H	M	H	H	H	N	N	H	H	H
LJ-Pv19100	Hyb	Shaanxi	N	N	H	H	H	H	L	H	H	H	N	N	H	H	H
LJ-Pv19111	Vv	Shaanxi	N	N	H	H	H	H	L	H	H	H	N	N	H	H	H
LJ-Pv19117	Vv	Shandong	N	N	H	H	H	H	H	H	H	H	N	N	M	H	H
LJ-Pv19119	Vv	Shandong	N	N	H	H	H	H	M	H	H	H	L	N	H	H	H
LJ-Pv19125	Hyb	Sichuan	N	N	H	H	H	H	H	H	H	H	N	N	H	H	H
LJ-Pv19128	Vv	Sichuan	N	N	H	H	H	M	M	H	H	H	N	N	H	H	H
LJ-Pv19131	Vv	Hunan	N	N	H	H	H	H	M	H	H	H	L	L	H	H	H
LJ-Pv19134	Vv	Xinjiang	N	N	H	H	H	H	L	H	H	H	L	N	H	H	H
LJ-Pv19135	Hyb	Guizhou	N	N	H	H	H	H	M	H	H	H	N	N	H	H	H
LJ-Pv19138	Vd	Guizhou	N	N	H	H	H	H	L	H	H	H	M	L	M	H	H
LJ-Pv19143	Vv	Gansu	N	N	H	H	H	H	N	H	H	H	L	L	H	H	H
LJ-Pv19146	Hyb	Gansu	N	N	H	H	H	H	M	H	H	H	N	N	H	H	L
LJ-Pv19150	Vv	Henan	N	N	H	H	H	H	M	H	H	H	N	L	H	H	H
LJ-Pv19154	Hyb	Henan	N	N	H	H	H	H	M	H	H	H	N	N	H	H	H
LJ-Pv19169	Hyb	Shanxi	N	N	H	H	M	H	L	H	H	H	N	N	H	H	H
LJ-Pv19177	Vv	Shanxi	N	N	H	H	H	H	L	H	H	H	L	L	M	H	H

N, not virulent; L, weakly virulent; M, moderately virulent; H: highly virulent. Isolates: The Pv isolates number. Host: the host of the Pv isolates. Location: the collection location of the Pv isolates. The hosts and collection sites of the Pv isolates were listed in the Supplementary Table S1.1. ^a^*M. rotundifolia* ‘Carlos’ (Mr). ^b^*M. rotundifolia* ‘Noble’ (Mr). ^c^*V. vinifera* ‘Thompson seedless’ (Vv). ^d^*V. vinifera* ‘Cabernet Sauvignon’ (Vv). ^e^*V. aestivalis* ‘SJTU118’ (Vae). ^f^*V. riparia* ‘SJTU127’ (Vri). ^g^*V. rupestris* ‘SJTU123’ (Vru). ^h^*V. davidii* ‘Huiliang’ (Vd). ^i^*V. davidii* ‘Ziqiu’ (Vd). ^j^*V. piasezkii* ‘SJTU001’ (Vp). ^k^*V. hancockii* ‘SJTU002’ (Vh). ^l^*V. amurensis* ‘Shuanghong’ (Vam). ^m^*V. yeshanensis* ‘SJTU004’ (Vy). ^n^*Vitis* interspecific crossing ‘Kyoho’ (Hyb). ^o^*Vitis* interspecific hybrid ‘Zuohong 1’ (Hyb). *Pv isolates collected from the wild grapevine in Changbai Mountain, Jilin province.

As expected, the Chinese isolates were non pathogenic to *M. rotundifolia* varieties and showed the highest virulence on *V. vinifera* varieties. Compared with three North American *Vitis* species, Chinese isolates were moderately virulent on *V. riparia* and *V. aestivalis*, while less virulent on *V. rupestris*. Among the Chinese *Vitis* species, Pv isolates were most virulent on *V. davidii* and *V. piasezkii*, followed by *V. yeshanensis,* and sometimes less virulent on *V. hancockii* and *V. amurensis.*

These Chinese isolates exhibited higher virulence on ‘Kyoho’ and ‘Zuohong 1’, which were expected as two interspecific hybrids with parentage traced back to Pv-susceptible *V. vinifera*. It was worth noting that virulence differed among these isolates when inoculated on the East Asian grape species *V. hancockii* and *V. amurensis*. Most isolates were isolated from Jilin Province, one of the origins and main growing areas of *V. amurensis*, exhibiting high virulence on *V. amurensis* and *V. hancockii*, whereas isolates from other regions exhibited low virulence on them.

### Interaction between the Chinese Pv isolates and grapevines

In the virulence test, the occurrence times and intensities of leaf necrosis were different in the interactions between Pv isolates and grapevines, suggesting that leaf necrosis might be used as an important indicator for the rapid identification of Pv resistance in grape germplasm. Therefore, histochemical staining was performed to observe the interaction between the Pv isolates and grapevines with different resistance levels.

**Figure 3:**
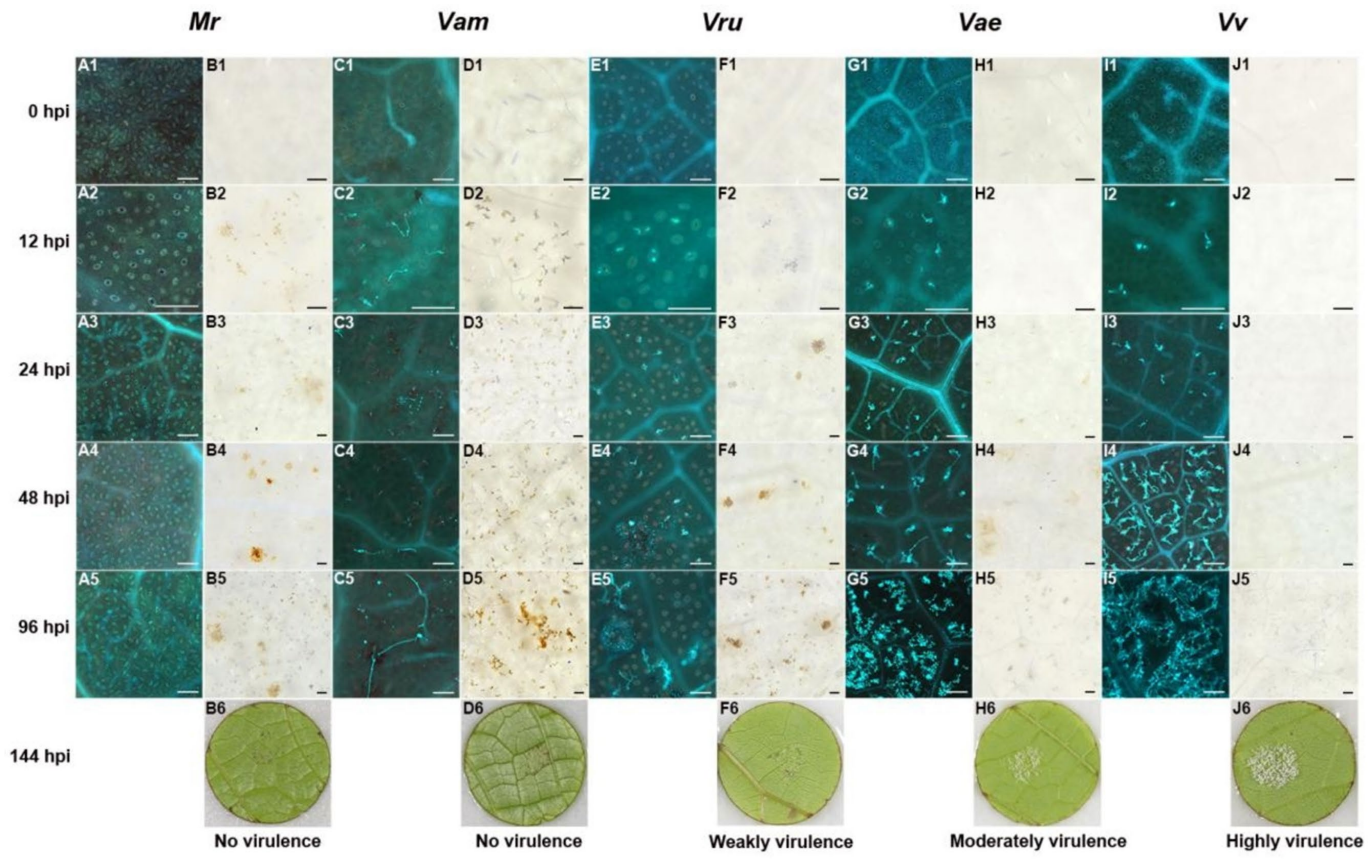
Histochemical observation of LJ-Pv18001 in grapevines. Histochemical staining was performed to detect cell death and Hypha growth at 0 hpi, 12 hpi, 24 hpi, 48 hpi, and 96 hpi, with trypan blue and aniline blue staining. A1 to 5, C1 to 5, E1 to 5, G1 to 5, and I1 to 5, the development of hyphae of LJ-Pv18001 in grapevines. B1 to 5, D1 to 5, F1 to 5, H1 to 5, and J1 to 5, the leaf necrosis caused by LJ-Pv18001 in grapevines. B6, D6, F6, H6, and J6, symptom of LJ-Pv18001 on grapevines at 144 hpi. Mr., *M. rotundifolia* ‘Carlos’. Vam, *V. amurensis* ‘Shuanghong’. Vru, *V. rupestris* ‘SJTU123’. Vae: *V. aestivalis* ‘SJTU118’. Vv, *V. vinifera* ‘Thompson seedless’. Bars = 100 um.

Hyphal growth was consistent with symptom development in grapevines. No hyphae were observed in Pv-immune *M. rotundifolia* ‘Carlos’ (Figure 3, A1 to A5), and only a few unbranched hyphae were observed in the highly resistant *V. amurensis* ‘Shuang Hong’ (Figure 3, C1 to C5). Some hyphae were observed in the moderately resistant *V. rupestris* ‘SJTU123’ (Figure 3 E1 to E5), whereas large number of hyphae were observed in the susceptible *V. aestivalis* ‘SJTU118’, although the elongation of the hyphae was impeded (Figure 3, G1 to G5). Dense and branched hyphae were observed in the highly susceptible variety *V. vinifera* ‘Thompson Seedless’ (Figure 3, I1 to I5).

**Figure 4:**
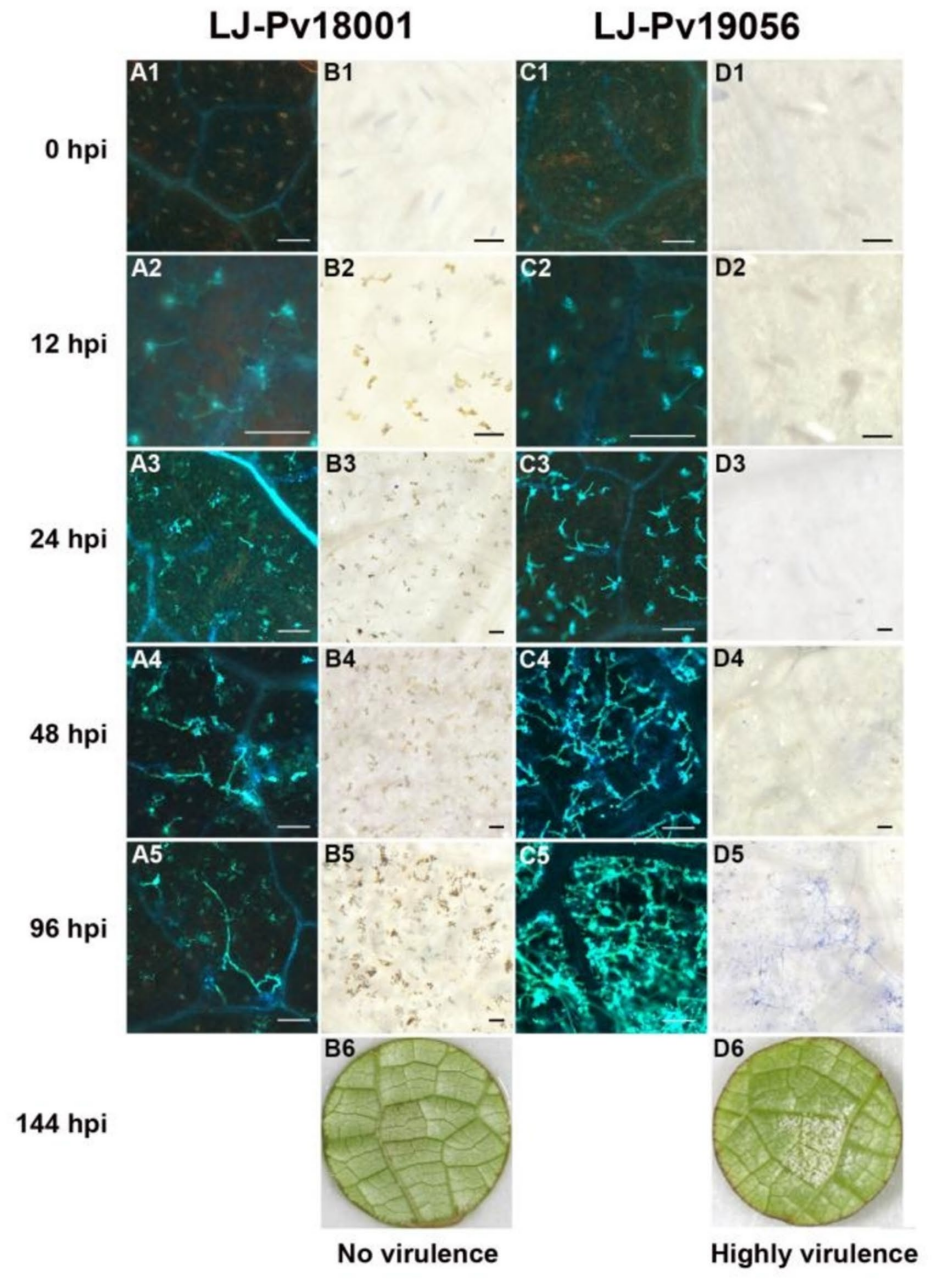
The development of *P. viticola* hyphae and the leaf necrosis in *V. amurensis* ‘Shuanghong’. Histochemical staining was performed to detect cell death and Hypha growth at 0 hpi, 12 hpi, 24 hpi, 48 hpi, and 96 hpi, with trypan blue and aniline blue staining. A1 to 5, the development of hyphae of LJ-Pv18001 in *V. amurensis* ‘Shuanghong’. C1 to 5, the development of hyphae of LJ-Pv19056 in *V. amurensis* ‘Shuanghong’. B1 to 5, the leaf necrosis caused by LJ-Pv18001 in *V. amurensis* ‘Shuanghong’. D1 to 5, the leaf necrosis caused by LJ-Pv19056 in *V. amurensis* ‘Shuanghong’. B6, D6, symptom of LJ-Pv18001 and LJ-Pv19056 in *V. amurensis* ‘Shuanghong’ at 144 hpi. VI, Virulence Index. Bars = 100 um.

Leaf necrosis produced through the hypersensitivity response (HR) is another obvious indicator reflecting the host resistance and incompatibility. The Pv-induced leaf necrosis was observed in highly resistant varieties at 12 hpi (Figure 3, B2, D2), and in moderately resistant grape variety at 24 hpi (Figure 3, F3), whereas that in susceptible grape variety was not observed until 48 hpi (Figure 3, H4). The leaf necrosis in highly resistant varieties was the most severe, followed by moderately resistant grape variety, and only slight leaf necrosis was observed in susceptible *V. aestivalis*. No necrosis was visible in highly susceptible *V. vinifera* (Figure 3, J1 to J5). In contrast to isolate LJ-Pv18001, isolate LJ-Pv19056 was compatible with *V. amurensis* varieties (Table 1). Compared with LJ-Pv18001, the hyphae of LJ-Pv19056 grew well on *V. amurensis* variety (Figure 4). These results demonstrated that both timing and severity of leaf necrosis varied among varieties with different resistance levels.

## Discussion

Downy mildew is one of the main diseases of grapevine in China. The study of the origins and genetic diversity of Pv populations in China may help to promote control measures to counteract downy mildew.

Phylogenetic analysis of 193 Pv isolates from 14 provinces in China at three loci (*Act*, *Tub*, and *Cytb*) revealed that all isolates belonged to *P. viticola* clade *aestivalis*, one of the five cryptic species of Pv in North America. Despite of the low bootstrap values of the ITS phylogenetic trees, the results of both MP and ML phylogenetic trees also supported this conclusion. Among these Pv isolates, 46 isolates (LJ-Pv19035 to LJ-Pv19080) collected from wild *V. amurensis* grapes in the natural habitat in Changbai Mountains also belong to *P. viticola* clade *aestivalis.* Considering the history of Pv invasion in Europe, and the subsequent spread to other viticulture areas along with distribution of grapevines, the finding that all Chinese Pv isolates belonged to the *P. viticola* clade *aestivalis* supports the hypothesis that Chinese Pv isolates originate from North America. Although all the Pv isolates collected from various viticultural regions and wild grape habitats in China belonged to the *P. viticola* clade *aestivalis*, other Pv cryptic species from North America may also exist in specific regions of China.

Zhang et al. (2017) reported that current Chinese Pv population is an admixture of endemic and introduced isolates. In their study, Chinese Pv population containing 103 isolates from 9 provinces were assigned to different cryptic species in different phylogenetic analyses (Zhang et al., 2017). The haplotype of the same cryptic species referring to Rouxel et al. (2013) and that in Zhang’s study were not clustered together, therefore incorrect parameters in Zhang’s phylogenetic analyses might be used. Phylogenetic trees based on the *Act* and *Tub* loci of these Chinese Pv isolates were also inferred (Supplementary Figure S2). Consistent with our results, these Chinese Pv isolates were classified as *P. viticola* clade *aestivalis*. These results support the long-standing hypothesis that Chinese Pv strains originated from North America. In other words, there is no evidence of the existence of native Pv populations in China.

The *Act* and *Tub* loci of Pv population isolated from China showed lower allelic diversity and nucleotide diversity than those of isolated from *P. viticola* clade *aestivalis* populations in North America according to Rouxel (Rouxel et al., 2013). Fontaine et al. (2021) analyzed the nucleotide diversity of 32 SSR loci and demonstrated that the *P. viticola* clade *aestivalis* population in North America had higher sequence diversity than other invasive populations, including Chinese population. The finding of lower diversity in Chinese populations supports the hypothesis that Pv was introduced to China. Although Pv is spread widely in China, the introduction of new strains or cryptic species will increase the population size and genetic diversity of Pv and hinder the control of the disease. Therefore, strict quarantine measures are still needed. Phylogenetic and sequence polymorphic analyses of the Chinese Pv population will be used to guide the quarantine work to prevent further invasion by other grapevine pathogens.

Quinone outside inhibitors (QoI) fungicides are widely used to prevent Pv infection, however, QoI fungicide-resistant Pv isolates have been found in many growing areas around the world (Chen et al., 2007; Delmas et al., 2017; Gisi and Sierotzki, 2008; Furuya et al., 2010; Sierotzki et al., 2005). These fungicides target cytochrome b of fungal mitochondria, in which a mutation of glycine at codon 143 (G143A) occurs and leads to the QoI fungicide resistance of Pv. In current study, a higher proportion of Pv isolates collected from vineyards contained G143A mutation compared with the Pv isolates collected in the wild. It suggests that directional natural selection is due to the widespread application of fungicides, which allows accumulation of fungicide-resistance mutations in commercial vineyards.

Pv produces oospores through sexual reproduction to survive in the winter. Therefore, heterozygosity was observed at the *Act* and *Tub* loci in some isolates, indicating that Pv outcrossing facilitated gene exchange among Pv individuals and adaptability to the environmental factors, including fungicides (Delmas et al., 2017) and host resistance (Peressotti et al., 2010). Heterozygous sites were found in 47 isolates collected from 10 provinces, including the northernmost province (Jinlin province) and the southernmost provinces (Guangxi province) of the sampling sites. It implies that outcrossing of Pv is widespread among the major growing areas in China.

Consistent with previous results on *P. viticola* clade *aestivalis* from North America (Rouxel et al., 2013), Chinese Pv isolates can infect a wide range of grapevine species. All Chinese Pv isolates showed similar virulence with North American varieties and Eurasian varieties. Chinese Pv isolates were weakly virulent on *V. rupestris* compared to North American isolates, except for the isolates collected from Jilin province which were weakly virulent on the Chinese wild species *V. amurensis* and *V. hancockii*. Even though native Pvs do not exist in East Asian area including China, some Pv-related species such as *Plasmopara cissii* and *Plasmopara amurensis* are supposed to co-evolve with their *Vitaceae* hosts in distribution area, causing the resistance of some East Asian grape species to Pv (Jürges et al., 2009; Dick, 2002; Grunzel, 1959).

The Eurasian species *V. vinifera* and its hybrids are cultivated worldwide. However, they are susceptible to Pv. It is a practical strategy to promote the resistance of Eurasian grapevine to Pv through the cross breeding between economic variety and resistant variety, rather than using fungicide (Merdinoglu et al., 2018; Töpfer and Trapp, 2022). Pv-resistant grapevine germplasm may promote the creation and deployment of Pv-resistant varieties in viticulture through cross-breeding. For example, pyramided plants containing *Rpv1* and *Rpv3* loci have showed complete resistance to Pv, compared to their parents (Nascimento-Gavioli et al., 2016). Therefore, breeding of resistant grape varieties and combination with fungicide treatments were effective long-term strategy for controlling grapevine downy mildew.

Histochemical staining suggest that leaf necrosis could be an important indicator for rapid identification of resistance to Pv in grape germplasm. A gene-for-gene model has been used to explain the incompatiblility interactions between the host and the pathogen (Flor, 1955). In this model, plant resistance proteins interact directly or indirectly with their corresponding pathogenic effector (avirulence) proteins, activating a hypersensitive defense response. In the present study, HR response was observed in grapevines inoculated with Pv isolates LJ-Pv18001. Leaf necrosis is the earliest phenotypic difference between resistant and susceptible grapevines (Bellin et al., 2009). A similar phenotype was also observed when resistant grapevines containing the *Rpv1* gene and susceptible grapevines were inoculated with Pv (Feechan et al., 2013). Similar phenomena have been reported in other plant pathosystems, such as *Erysiphe necator* with grapevines (Feechan et al., 2011), *Hyaloperonospora parasitica* with *Arabidopsis* (Coates and Beynon, 2010), and *Puccinia glumarum* with wheat (Stakman, 1915). HR response varies in occurrence time and intensity (magnitude) following the grapevine variety and Pv isolate combination. This was likely due to differences in grapevine resistance genes and Pv effector (avirulence) genes, however, it remains to be explored in future studies.

Overall, these results demonstrate that Chinese Pv isolates originate from North America, belonging to *P. viticola* clade *aestivalis* and infecting a wide range of hosts. Analysis of the evolution and pathogenicity of Pv isolates in China, as well as the study of the interaction between Pv and different resistant grapevine varieties, will aid the design of quarantine regulations and high-efficiency resistance breeding to prevent grapevine downy mildew.

## Data Availability

The datasets presented in this study can be found in online repositories. The names of the repository/repositories and accession number(s) can be found in the article/Supplementary material.

## Footnotes

^1^ http://www.foooooot.com/trip/2865547/

^2^ https://www.oiv.int/node/2830
